# Real-world﻿ artificial intelligence-based opportunistic screening for diabetic retinopathy in endocrinology and indigenous healthcare settings in Australia

**DOI:** 10.1038/s41598-021-94178-5

**Published:** 2021-08-04

**Authors:** Jane Scheetz, Dilara Koca, Myra McGuinness, Edith Holloway, Zachary Tan, Zhuoting Zhu, Rod O’Day, Sukhpal Sandhu, Richard J. MacIsaac, Chris Gilfillan, Angus Turner, Stuart Keel, Mingguang He

**Affiliations:** 1 Centre for Eye Research Australia, Royal Victorian Eye and Ear Hospital, Level 7, 32 Gisborne Street, East Melbourne, VIC 3002 Australia; 2 Melbourne School of Population and Global Health, University of Melbourne, Melbourne, Australia; 3 School of Psychology, Deakin University, Geelong, VIC Australia; 4 The Australian Centre for Behavioural Research in Diabetes, Diabetes Victoria, Melbourne, VIC Australia; 5 Department of Ophthalmology, Guangdong Academy of Medical Sciences, Guangdong Provincial People’s Hospital, Guangzhou, China; 6 Department of Endocrinology and Diabetes and Department of Medicine, St Vincent’s Hospital Melbourne, Fitzroy, Australia; 7 Department of Endocrinology, Eastern Health, Melbourne, Australia; 8 Lions Eye Institute, 2 Verdun St, Perth, Australia; 9 Ophthalmology, Department of Surgery, University of Melbourne, Melbourne, Australia

**Keywords:** Imaging, Eye diseases, Machine learning

## Abstract

This study investigated the diagnostic performance, feasibility, and end-user experiences of an artificial intelligence (AI)-assisted diabetic retinopathy (DR) screening model in real-world Australian healthcare settings. The study consisted of two components: (1) DR screening of patients using an AI-assisted system and (2) in-depth interviews with health professionals involved in implementing screening. Participants with type 1 or type 2 diabetes mellitus attending two endocrinology outpatient and three Aboriginal Medical Services clinics between March 2018 and May 2019 were invited to a prospective observational study. A single 45-degree (macula centred), non-stereoscopic, colour retinal image was taken of each eye from participants and were instantly screened for referable DR using a custom offline automated AI system. A total of 236 participants, including 174 from endocrinology and 62 from Aboriginal Medical Services clinics, provided informed consent and 203 (86.0%) were included in the analysis. A total of 33 consenting participants (14%) were excluded from the primary analysis due to ungradable or missing images from small pupils (n = 21, 63.6%), cataract (n = 7, 21.2%), poor fixation (n = 2, 6.1%), technical issues (n = 2, 6.1%), and corneal scarring (n = 1, 3%). The area under the curve, sensitivity, and specificity of the AI system for referable DR were 0.92, 96.9% and 87.7%, respectively. There were 51 disagreements between the reference standard and index test diagnoses, including 29 which were manually graded as ungradable, 21 false positives, and one false negative. A total of 28 participants (11.9%) were referred for follow-up based on new ocular findings, among whom, 15 (53.6%) were able to be contacted and 9 (60%) adhered to referral. Of 207 participants who completed a satisfaction questionnaire, 93.7% specified they were either satisfied or extremely satisfied, and 93.2% specified they would be likely or extremely likely to use this service again. Clinical staff involved in screening most frequently noted that the AI system was easy to use, and the real-time diagnostic report was useful. Our study indicates that AI-assisted DR screening model is accurate and well-accepted by patients and clinicians in endocrinology and indigenous healthcare settings. Future deployments of AI-assisted screening models would require consideration of downstream referral pathways.

## Introduction

Over 430 million people are living with diabetes globally^1^, with retinopathy a common diabetic complication. According to projected changes in population and prevalence, the number of people that will require routine retinal screening for diabetic retinopathy (DR) is set to almost double over the next two decades^2^. This is likely to have significant demand implications for global ophthalmic workforces which are already under-resourced^3^.

Screening for DR within non-ophthalmic settings has been adopted globally and has had varying success in increasing accessibility and early detection of disease^4–6^. National DR screening programs have been shown to be successful in settings such as the United Kingdom (UK), where DR has been managed such that it is no longer the leading cause of blindness in working aged adults^6^. In Australia however, adherence to recommended DR screening guidelines has been reported to be as low as 77.5% in non-Indigenous, and 52.7% in Indigenous populations^7^. The prevalence of diabetes is up to six times higher in Indigenous Australians and the development of late-stage DR significantly more likely within this at-risk population^8^. In response to the growing prevalence of diabetes-related vision loss, the Australian Government has introduced a Medicare reimbursement code for non-eye health care professionals to perform DR screening. Nevertheless, uptake of this new care model has been sub-optimal, in part due to non-eye health care professionals lacking the time and confidence to detect retinal features associated with DR^9^.

Artificial intelligence (AI)-assisted systems provide a potential solution by enabling quick and accurate assessment of the retina at the point-of-care. Automated analysis of retinal images using AI has been shown to be highly accurate using various retinal camera models, imaging protocols, and across multiple ethnicities^10–13^. A major criticism however has been the lack of validation in real-world settings where accuracy is likely to be compromised due to variations in disease prevalence, image quality, and patient characteristics^14,15^. International experience with the real-world use of AI-based DR screening systems is early and emerging^16–18^, and there remains an overall paucity of evidence which explores the factors that underlie the successful implementation of these systems.

Engaging with end-users (patients, clinicians, and organisational stakeholders) to explore their experiences and satisfaction with AI-assisted systems will be important in developing novel AI-assisted screening models that are well-utilised. The widespread adoption of this promising new technology in routine clinical practice will require consideration and integration of the system’s capabilities and design within clinical workflow contexts^19^. Further, there is a paucity of evidence which explores the real-world accuracy of AI-assisted DR screening systems, and the satisfaction of end-users who have had firsthand experience with this technology^20^.

The purpose of this study was thus to implement an opportunistic AI-assisted DR screening model in real-world settings, including in endocrinology outpatient and Aboriginal Medical Service clinics. The diagnostic performance, feasibility, and end-user experiences of this novel model were subsequently evaluated.

## Methods

This study employed a mixed methods approach to evaluate the feasibility, acceptability, and accuracy of a novel AI-assisted DR screening model. The study consisted of two components: (1) a prospective observational study using an AI-assisted system for patient DR screening, and (2) in-depth interviews with health professionals involved in screening implementation.

### Real-world AI screening

#### Participants

Two endocrinology outpatient clinics in Melbourne (Box Hill Hospital and St Vincent’s Hospital) and three Aboriginal medical service clinics in metropolitan Western Australia (Derbarl Yerrigan Health Service [DYHS]) participated in this study. The Western Australian clinics are dedicated to caring for Aboriginal people and include a walk-in ophthalmology clinic in East Perth, and two general practitioner (GP) clinics (Maddington and Mirrabooka). Patients across all clinics aged over 18 years with type 1 (T1DM) or type 2 diabetes mellitus (T2DM) were invited to participate in the present study. Patients were excluded if they were unable to give informed consent. Reasons for refusal were recorded for those who declined to participate. Recruitment occurred across all sites between March 2018 and May 2019.

Ethical approval was obtained from the Royal Victorian Eye and Ear Hospital (16-1268H), St Vincent’s Hospital Melbourne (LNR/17/SVHM/39), Eastern Health (LR95/2017) and the Western Australian Aboriginal Health (846) Human Research Ethics Committees. This study adhered to the tenets of the Declaration of Helsinki and all participants provided written informed consent.

#### Automated artificial intelligence system development

The offline automated AI system utilised in this study (i.e. the index test) simultaneously screened for diabetic retinopathy (DR), age related macular degeneration (AMD), and glaucoma. The development and validation of each algorithm has been described in extensive detail previously^11,21,22^. Briefly, each algorithm was trained using over 200,000 retinal images acquired from a web-based, cloud sourcing platform (www.labelme.org). These retinal images were collected from ophthalmology departments, optometry clinics, and screening settings in China using various retinal camera models (Topcon, Canon, Heidelberg and Digital Retinography System). The presence of ocular disease was determined by 21 ophthalmologists who were able to achieve a substantial level of intra-observer agreement (Kappa ≥ 0.70) on a test set of retinal images. Referable DR was defined as ≥ pre-proliferative DR and/or diabetic macular oedema (DMO) using the National Health Screening (NHS) diabetic eye screening guidelines^23^. At the completion of a grading, images were randomly assigned to either a training or validation dataset. Deep learning models were developed for each disease, all using the Inception-v3 architecture. These included: (1) classification for disease, (2) assessment of image quality, (3) assessment of image quality, and presence of the macular region for determining DMO.

#### Testing protocol

Prior to commencement of patient recruitment, staff (including nurses, optometrists, Aboriginal health workers) from participating clinics who expressed interest in DR screening were given one-on-one training by the same trainer (JS). Training consisted of recruitment and consenting procedures, an overview of ocular pathology, visual acuity (VA) testing, acquisition of retinal images, troubleshooting techniques for poor quality images, automated grading using the offline AI system, and delivery of grading report to patients. Training and supervision were provided until staff were comfortable with performing data collection and imaging independently.

**Figure 1:**
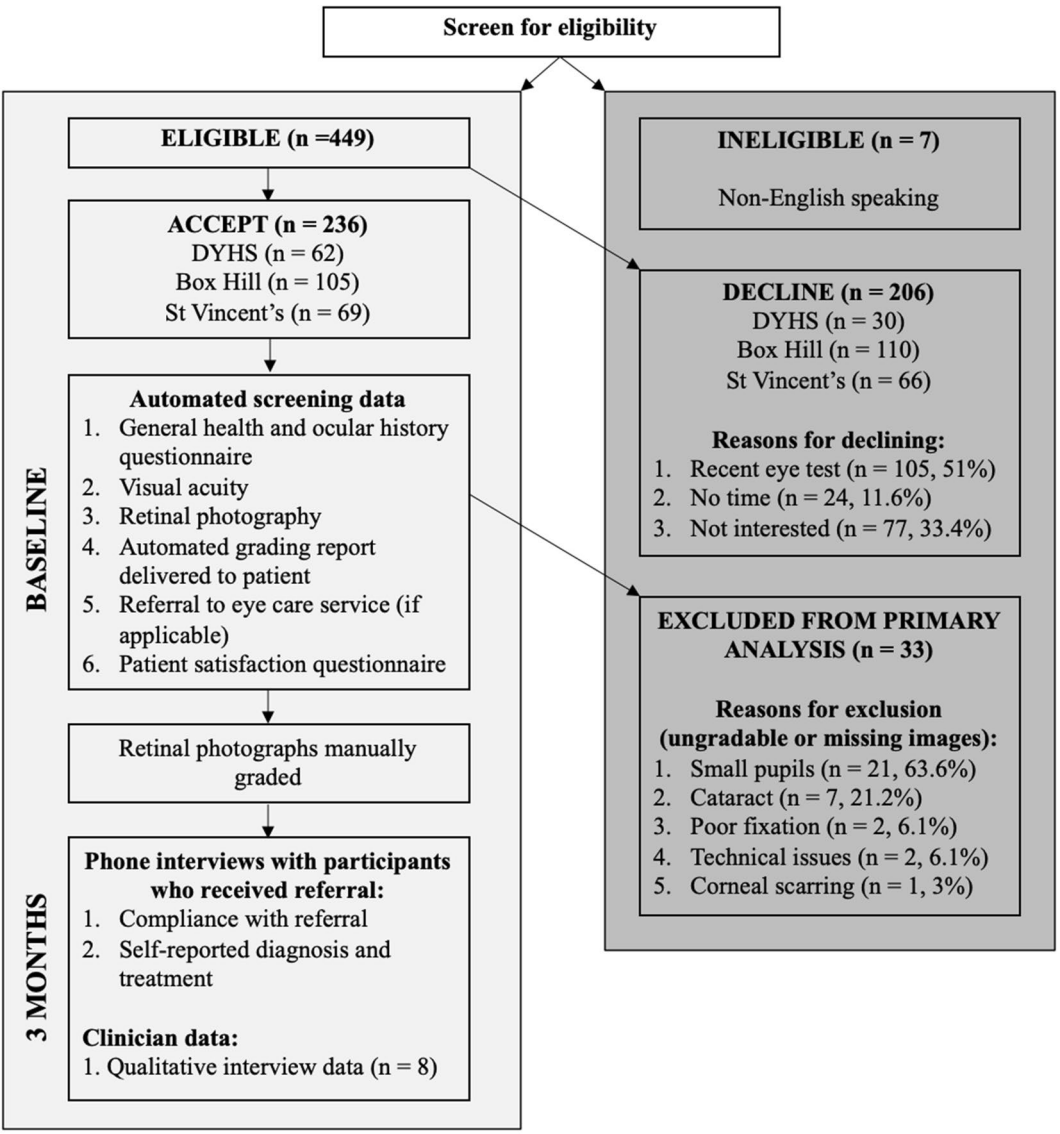
Flow chart of screening procedures and follow-up of participants and screening staff.

**Figure 2:**
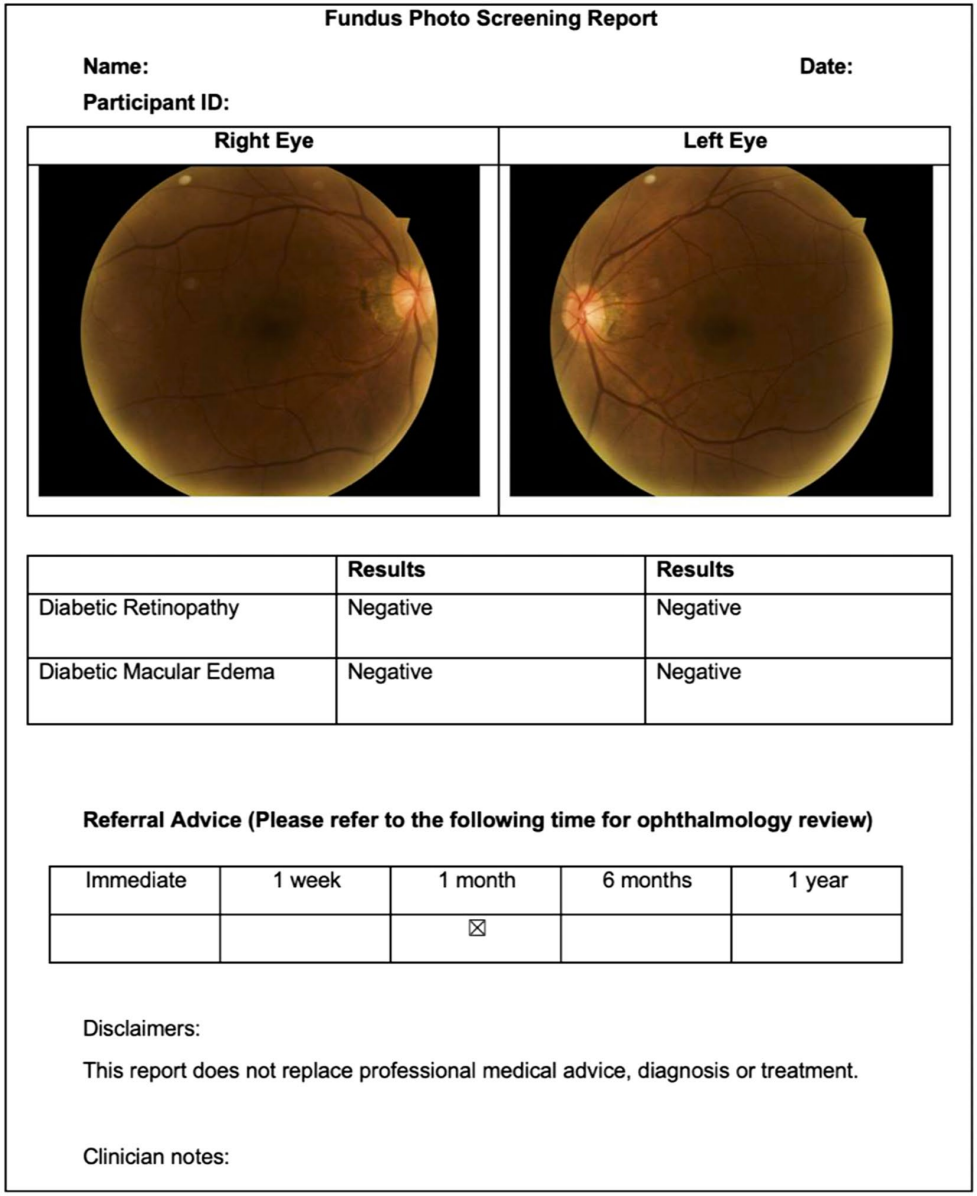
Sample grading report generated by artificial intelligence-based screening system, including diabetic retinopathy status and referral recommendations. Details of the development and validation of this artificial intelligence system have been described previously^11,21,22^.

Study procedures from eligibility screening to final manual grading of retinal images are summarised in Fig. 1. Information on general health, sociodemographic factors, previous ocular history, and time since last eye exam were collected from all eligible and consenting participants using a paper-based questionnaire. Distance VA was measured for each eye using a standard 3-m logMAR chart in a well-lit room. Participants were tested with correction if they routinely wore glasses for distance tasks. Following this, a single 45-degree (macula centred), non-dilated, non-stereoscopic, colour retinal image was taken of each eye by clinic staff using the Digital Retinography System camera (DRS, CenterVue SpA, Italy) at St Vincent’s Hospital Melbourne, DYHS Maddington and Mirrabooka; the Canon CR-2 AF camera (Canon, Tokyo, Japan) at Box Hill Hospital; and the Topcon 3D OCT1 Maestro camera (Topcon, Tokyo, Japan) at DYHS East Perth. Imaging was repeated if poor quality images were obtained on the first attempt. Retinal images of participants were immediately uploaded to an offline version of the automated AI system for grading. A grading report was generated and printed for participants at the time of screening, which included DR status and referral recommendations (Fig. 2). Participants who were positive for referable DR, age-related macular degeneration, or glaucoma if previously undiagnosed; or had a VA of < 6/12 (20/40) in either eye (i.e. the Australian driving standard) received a referral to an optometrist or ophthalmologist. Following automated screening, participants completed a modified version of the Client Satisfaction Questionnaire (CSQ-8) to collect data on overall satisfaction, and how likely they were to use the AI screening service again^24^.

#### Reference standard grading

To determine the accuracy of the AI screening model, all retinal images were manually graded for referable DR by two NHS-certified retinal graders using the NHS diabetic eye screening guidelines^23^. As the reference standard, referable DR was diagnosed if pre-proliferative DR or worse (equivalent to an Early Treatment Diabetic Retinopathy Study Severity Scale level of ≥ 43) and/or DMO was detected in either eye on manual grading. Any disagreements were adjudicated by two retinal specialists (SS and RO). Participants who were subsequently identified as false negatives (i.e. no DR reported by the AI system for either eye, but DR subsequently detected on manual grading) were contacted via telephone and advised to visit an optometrist or ophthalmologist.

#### Telephone follow up

Participants who received a referral were contacted via telephone three months after their baseline assessment to determine compliance with referral, and self-reported diagnosis among those who visited an ophthalmologist. Three attempts were made to contact participants to obtain this information.

#### Data analysis

All study data were manually entered and managed using REDCap electronic data capture tools which were hosted at the Centre for Eye Research Australia (CERA). De-identified data were downloaded from REDCap and imported into Stata/IC version 15.1 (College Station, Texas, USA) for statistical analysis.

Participants with missing reference standard grading (due to bilateral missing images, or both eyes ungradable on manual grading), and those with missing/ungradable images from one eye and no referable DR detectable in the opposite eye on manual grading were excluded from analysis. Participants with missing/ungradable images from one eye and referable DR detected in the fellow eye on manual grading were included in the analysis and classified as having referable DR. As specified *a* *priori*, participants with retinal images from either eye reported to be ungradable by the offline AI system were categorised as having a positive index test for referable DR, to reflect current Australian referral guidelines which state that patients should be referred if the fundus is not able to be viewed during screening^25^.

Sensitivity, specificity, area under the curve (AUC), positive predictive value (PPV) and negative predictive value (NPV) were estimated with 95% exact binomial confidence intervals (CI) to evaluate diagnostic accuracy. A sensitivity analysis including all participants was performed to explore the potential impact of selection bias introduced by excluding participants with missing reference standard diagnosis. In this sensitivity analysis, diagnostic accuracy parameters were first estimated with the reference standard for the missing/ungradable images classified as positive for referable DR (worst-case scenario), and then with the reference standard for the missing/ungradable images classified as negative (best-case scenario)^26,27^. A sub-group analysis was performed according to diabetes type (T1DM vs T2DM), clinic type (endocrinology vs Aboriginal medical service), and camera model (Canon, DRS and Topcon).

### Health professional in-depth interviews

#### Study design and participants

Medical professionals and allied health screening staff at participating sites were invited to take part in one-on-one interviews at the conclusion of participant recruitment. Interviews were conducted between December 11, 2018 and September 5, 2019 by an experienced qualitative researcher (EH) who had no prior relationship with the recruitment sites or involvement in the development of the AI system. Interviews were conducted via videoconference or face-to-face; in a private setting within the participants’ workplace or CERA. The interview guide was semi-structured to prompt consideration of potential barriers and facilitators to using the AI system, whilst allowing flexibility for participants to contribute their own views. The interview questions were developed by conducting a thorough search of the literature and were informed by the Consolidated Framework for Implementation Research^28^. The research team (EH, JS, SK, MH) provided input into the development of the questions. Interviews explored medical professional and allied health screening staff experiences with the AI system, factors most important for implementation into clinical practice, and the advantages of AI in healthcare settings.

#### Data analysis

The qualitative analysis was performed by two research fellows (EH and JS, both experienced qualitative researchers). The interviews were audio-recorded, transcribed verbatim, anonymised, and imported into NVivo V.12 (QSR International, Melbourne, Australia) to facilitate data management. The data were analysed using inductive ‘bottom-up’ thematic analysis^29^. Analysis commenced with EH familiarising themselves with the data and generating an initial coding framework, grounded in the data. JS reviewed the data and coding and confirmed that the analysis was credible, and that themes were grounded in the data. Codes were then built into broader categories through comparison across transcripts and higher-level recurring themes were developed. Emergent themes were discussed by two researchers (EH & JS) until consensus was reached. Reliability was ensured through continuous discussion about the data with the research team. The number of times (frequency) each key theme was reported by participants was computed and summarised in the results.

## Results

A total of 456 participants were invited to participate in this study, of whom 236 (51.8%) consented and were screened for disease (Fig. 1). Reasons for declining across each clinic can be found in Fig. 1, including 105 (51.0%) who had a recent eye test completed, 24 (11.6%) who had no time, and 77 (33.4%) who were not interested.

33 consenting participants (14%) were excluded from the primary analysis. Reasons for ungradable or missing images were small pupils (n = 21, 63.6%), cataract (n = 7, 21.2%), poor fixation (n = 2, 6.1%), technical issues (n = 2, 6.1%), and corneal scarring (n = 1, 3%). Excluded participants were more likely to be male (58%), older (median 65 years of age), have T2DM (88%), and had been diagnosed diabetic for longer (median 20 years) than those included in the primary analysis (Table 1).

Among the 203 participants included in the primary analysis, 101 were female and 102 were male. The median (IQR) age of study participants was 56 (40–67) years. The median (IQR) duration of diabetes diagnosis was 13 (5–20) years and 130 (65%) had type 2 diabetes. More than half of participants (n = 106, 56%) reported that it had been more than 12 months since their last diabetic eye examination. Referable DR was detected in 30 participants (15%) on manual grading. A summary of participant characteristics can be found in Table 1.

**Table 1:** Characteristics of participants included and excluded from primary analysis.

Characteristic	Complete case set				Excluded n = 33
	Box Hill Hospital n = 93	St Vincent’s Hospital, Melbourne n = 59	Derbarl Yerrigan n = 51	Total n = 203	
Sex, n (%)					
Male	46 (49%)	34 (58%)	22 (43%)	102 (50%)	19 (58%)
Female	47 (51%)	25 (42%)	29 (57%)	101 (50%)	13 (42%)
Age (years)^a^, median (IQR)	51 (38–67)	46 (29–63)	62 (56–67)	56 (40–67)	65 (58–78)
Diabetes type^a^, n (%)					
Type 1 diabetes	36 (39%)	34 (58%)	0 (0%)	70 (34%)	4 (12%)
Type 2 diabetes	55 (61%)	25 (42%)	51 (100%)	130 (65%)	29 (88%)
Diabetes duration (years), median (IQR)	10 (4–20)	14 (5–23)	15 (10–20)	13 (5–20)	20 (7–29)
Last eye exam^a^, n (%)					
≤ 12 months	61 (75%)	26 (63%)	27 (74%)	134 (71%)	18 (56%)
≥ 12–24 months	17 (21%)	15 (26%)	10 (20%)	42 (22%)	12 (38%)
> 24 months	3 (4%)	6 (11%)	3 (6%)	12 (7%)	2 (6%)
Referable DR, n (%)					
No	86 (92%)	55 (93%)	32 (63%)	173 (85%)	–
Yes	7 (8%)	4 (7%)	19 (37%)	30 (15%)	–

IQR = interquartile range; DR = diabetic retinopathy.

^a^Missing data for age (n = 1 included, n = 2 excluded), diabetes type (n = 2 included), time since last eye exam (n = 15 included, n = 1 excluded).

### Diagnostic accuracy

The AUC, sensitivity, and specificity of the offline AI system for referable DR across all clinics were 0.92, 96.9%, and 87.7% respectively (Table 2). In total, there were 51 disagreements (including 29 which were manually graded as ungradable) between the reference standard and index test diagnoses (Table 3). 21 of these were false positives and there was one false negative.

**Table 2:** Diagnostic accuracy for referable diabetic retinopathy.

	Primary analysis (n = 203)	Sensitivity analysis scenario (n = 236)	
	Complete-case set (95% CI)	Best-case (95% CI)	Worst-case (95% CI)
Sensitivity (%)	96.9 [83.8–99.9]	93.5 [78.6–99.2]	85.9 [75.0–93.4]
Specificity (%)	87.7 [81.8–92.2]	76.1 [69.7–81.8]	86.6 [80.6–91.3]
Area under the curve	0.92 [0.88–0.96]	0.85 [0.80–0.90]	0.86 [0.81–0.91]
Predictive value			
Positive (%)	59.6 [45.1–73.0]	–	–
Negative (%)	99.3 [96.4–100]	–	–

CI, confidence interval.

**Table 3:** Cross tabulation results between artificial intelligence system and manual grading.

Manual grading	AI grading			Total
	Non-referable DR	Referable DR	Ungradable	
Non-referable DR	150	21	0	171
Referable DR	1	31	0	32
Ungradable	7	22	4	33
Total	158	74	4	236

DR = diabetic retinopathy.

Sensitivity analyses suggested that sensitivity could be as low as 85.9% and specificity could be as low as 76.1% under the worst- and best-case scenarios, respectively (Table 2). Sub-group analysis showed higher diagnostic accuracy amongst those with T1DM compared to those with T2DM and for those seen at endocrinology clinics compared to those seen at Aboriginal medical services clinics (Table 4). Diagnostic accuracy was lower for the Topcon Maestro camera compared to the DRS and Canon (Table 4).

**Table 4:** Sub-group analysis (n = 203).

Subgroup	Sensitivity (%, 95% CI)	Specificity (%, 95% CI)	AUC (%, 95% CI)
*Diabetes type*			
Type 1	100.0 [39.8–100.0]	90.9 [81.3–96.6]	0.96 [0.92–0.99]
Type 2	96.4 [81.7–99.9]	85.3 [76.9–91.5]	0.91 [0.86–0.96]
*Clinic*			
Endocrinology	100.0 [71.5–100.0]	91.5 [85.6–95.5]	0.96 [0.93–0.98]
Aboriginal medical service	95.2 [76.1–99.9]	70.0 [4.86–81.4]	0.83 [0.73–0.92]
*Camera model*			
Canon	100.0 [59.0–100.0]	90.7 [82.5–95.9]	0.95 [0.92–0.98]
DRS	100.0 [63.1–100.0]	91.4 [82.3–96.8]	0.96 [0.92–0.99]
Topcon Maestro	94.1 [71.3–99.9]	53.3 [26.6–78.7]	0.74 [0.59–0.88]

### Participant referral and follow up

A total of 28 participants were given a referral based on new ocular findings identified during screening (referable DR = 10, VA < 6/12 = 4, ungradable images = 9, age-related macular degeneration = 1, and glaucoma = 4). Only 15 (53.6%) were able to be contacted to determine adherence to referral and self-reported diagnosis. Of these, 9 (60%) adhered to their referrals and attended an optometry or ophthalmology service for review. Of the five contacted participants who were referred for DR or ungradable images, two were diagnosed with referable DR, one was diagnosed with cataract, and three had no apparent abnormalities detected on ophthalmic examination. Of the four participants examined following referral for other reasons, one was diagnosed with early macular changes, two with refractive error, and one had no apparent abnormalities. Reasons for not visiting an eye care provider included: forgot they were told to have a follow-up (n = 5); no time (n = 2); had been overseas for an extended period of time (n = 1); and financially unable (n = 1).

### Participant satisfaction

Complete questionnaire data were available for 207 participants. Of these, 93.7% (194/207) were either satisfied or extremely satisfied with the automated retinal screening model. Overall, 93.2% (193/207) of participants specified they would be likely or extremely likely to use the service again. Two participants indicated they were very dissatisfied and two were unlikely to use the service again. These participants did not provide a reason for their dissatisfaction with automated screening.

### Staff training

On average, three hours of one-on-one training and four supervised clinic sessions were required for clinic staff to become competent and confident in performing the protocol procedures.

### Staff satisfaction and experiences with the automated screening model

All invited staff consented to participate in qualitative interviews. Medical professionals (endocrinologists = 2; ophthalmologists = 1) were all male and had a mean (SD) of 17.7 (7.5) years of clinical experience. Allied health screening staff (nurses = 3; optometrists = 1; and Aboriginal health workers = 1) were all female and had a mean (SD) of 16.4 (8.8) years of clinical experience. The interviews ranged from 15 to 60 min in length, with most lasting approximately 30 min.

### Aspects of the AI screening model that worked well

Both medical professionals and allied health screening staff reported that the AI system was easy to use (theme frequency = 14; Supplementary Table 1). The training and upskilling of staff in the use of retinal cameras and the AI software was also perceived as a positive aspect of the AI system. Staff perceived the real-time report generated to be advantageous for both patients and eye health professionals (theme frequency = 9) and that it was easy to interpret (theme frequency = 9).

### Challenges experienced with the AI screening model

The main challenge (Supplementary Table 2) reported by both medical professionals (theme frequency = 7) and allied health screening staff (theme frequency = 18) was the use of the AI system in the context of the research study. For example, recruiting patients and gaining informed consent added additional time and burden to the screening process, within a busy clinic. Staff reported some difficulties taking retinal photographs due to patient factors (theme frequency = 13), such as small pupil size. Staff also reported that a lack of knowledge regarding retinal imaging (and eye health more broadly) (theme frequency = 9), and informing patients about abnormal features the AI system had detected, as a barrier to use. This impacted upon staff confidence in discussing results with patients. Medical professionals (theme frequency = 3) and allied health screening staff (theme frequency = 4) reported limitations with the system setup in several locations. Transferring the images from the camera to the AI system added some burden to a process that was otherwise described as quick and easy. Staff also reported that a lack of available opportunity to use the system (due to low level of recruitment) was a barrier to use (theme frequency = 7).

### Factors important for implementing AI into clinical practice

Participants reported several factors important for implementing this technology within clinical settings (Supplementary Table 3). For screening staff (theme frequency = 17), having an efficient and user-friendly set-up of the AI system was the most important factor for implementation. This included an integrated or single-step process for capturing retinal images and running the AI software. For medical professionals (theme frequency = 10), the most important factor was having a clear referral pathway, ensuring that patients received appropriate follow-up care in response to their results. Ongoing staff training in the use of the system, as well as additional training in eye health and retinal imaging was perceived by screening staff as important for implementation (theme frequency = 11).

### Acceptance and/or trust of AI systems

Both medical professionals (theme frequency = 8) and allied health screening staff (theme frequency = 3) reported that evidence to support the validity and reliability of AI systems would enhance acceptance and trust in these systems, such as the one utilised in the current study. Screening staff (theme frequency = 3) also reported that having the results validated by a clinician would increase their confidence in the system.

### Value of AI screening for improving eye health

Both medical professionals (theme frequency = 6) and allied health screening staff (theme frequency = 10) reported that the AI system had an important place in eye health as a tool for enhancing the detection and diagnosis of eye conditions, particularly to meet the demands of the increasing number of patients who require screening for eye conditions (Supplementary Table 4). The AI system was also perceived to reduce the burden on eye health professionals and enhance the efficient utilisation of eye care services (medical professionals theme frequency = 7).

## Discussion

Using a mixed methods approach, this study prospectively evaluated the diagnostic accuracy of an opportunistic AI-assisted DR screening model in endocrinology and Aboriginal medical services clinics. The AI-assisted DR screening model achieved high accuracy, while compliance with referral was moderate. To our knowledge, this study is one of the first to date globally to investigate the experiences and acceptance of AI from the perspective of patients, clinicians, and organisational stakeholders.

The primary outcome for this study of diagnostic accuracy was high, indicating that the overall performance of our offline AI system is robust in real-world settings. Previous studies investigating the diagnostic performance of AI systems for the detection of referable DR using retrospective datasets have also demonstrated high levels of accuracy^10,11,13,30^. The real-world performance of these algorithms is essential to assess the impact of varying image quality and system usability. Real-world performance has been reported to range from being marginally less accurate, to similar and high in accuracy when compared against validation using high quality retrospective datasets^12,14,16–18,20,31,32^.

It is promising that our system has exceeded sensitivity and specificity endpoints set by the United States Food and Drug Administration (FDA) (> 85% and > 82.5% respectively) for the evaluation of AI-based DR screening systems. This is despite our study sample size being considerably smaller than that of the pivotal trial for the IDx-DR system which led to its regulatory approval. We note however that these endpoints were previously set for AI based on 7-field ETDRS photography, compared to the single field approach adopted in this study^12^. A similar real-world study utilising healthcare workers and an offline algorithm for the detection of DR using images captured using a non-mydriatic smartphone camera, has also been shown to exceed these pre-defined thresholds, achieving a sensitivity of 100% and specificity of 88.4%^33^. Whilst there are advantages to using a portable smartphone camera, it is possible that disease features (e.g. microaneurysms, hard exudates etc.) may be missed by this AI system and manual graders; due to the lower resolution of captured images compared to high-quality desktop fundus cameras, which may also lead to overly-high reported levels of sensitivity.

Sub-group analysis revealed that specificity was substantially lower in participants recruited from Aboriginal medical services and those imaged used the Topcon Maestro camera. Previous validation of the algorithm utilised in this study found a marginally lower AUC in a dataset of Indigenous images compared to those of Chinese, Malay and Caucasian ethnicity^11^. Indigenous images from the external validation dataset were captured using a Canon camera, indicating that the camera model is less likely to have caused this lower specificity. Many misclassified images were of poorer quality (shadowing from eyelashes or lens irregularities), possibly due to higher median age and rates of co-existing eye disease in those recruited from Aboriginal medical services clinics; leading to disagreement between the AI system and manual graders. Further, the AI system was trained exclusively using images from Chinese adults. Differences in retinal pigmentation may have some impact on the accuracy of the system. Additional system training and stricter re-imaging protocols are possible solutions to enable improved specificity in the Indigenous population.

Unlike many other studies investigating AI performance, our study included patients with both T1DM and T2DM. Of the few reported studies that have included T1DM patients, none performed a separate analysis based on disease type^12^, and in one case, disease type was missing in approximately 70% of cases^34^. Although the prevalence of T2DM is significantly higher, those with T1DM often have more severe ocular complications due to earlier onset of disease, making it critical to assess performance by disease type in this cohort. Results showed that diagnostic performance was better in those with T1DM compared to those with T2DM. This finding is encouraging given that our AI system was trained using images from Chinese adults aged over 40 years, who are unlikely to exhibit retinal reflections from the internal limiting membrane which are commonly seen in younger patients^35^, and have the potential to cause classification errors. Although this study did not collect sufficient data to draw conclusive findings on T1DM patients, these preliminary data may serve as the basis of future studies that focus on T1DM.

The implementation of an opportunistic AI-assisted DR screening model has the potential to greatly improve detection rates and adherence to referral. Participants were informed both verbally and via a printed report of any positive findings. However, almost half did not adhere to recommendations to see an optometrist or ophthalmologist for review. Of note, those who had eye examinations in the previous 12 months were not excluded from participation. In real-world settings, only those who are not meeting screening recommendations would be offered an examination, and this is the likely reason for the low percentage of ‘new’ referral cases. Disease management and referral adherence have been shown to improve when diabetic patients are provided with appointment letters and are sent mobile SMS reminders about upcoming reviews^36,37^. The low rate of adherence to referral highlights the importance of establishing a referral pathway that is effective and well-coordinated, before new screening models are introduced. Placing the onus on screening clinics to make follow-up appointments for patients will likely increase adherence and lead to better visual outcomes for patients.

The uptake of AI will largely be driven by clinician acceptance and whether the technology adds perceived value to patient care. Our current and previous pilot efforts^20^ have shown that patients are accepting of this technology, and the vast majority expressed willingness to undergo future testing again using the AI-assisted screening model. Surveys investigating clinician perceptions of AI and how they impact on workforce needs are an emerging research focus. Recent attention has been on medical specialities which utilise image analysis for diagnosis, and most have found that clinicians believe that the introduction of AI will have a positive impact^38–40^. However, these surveys have not explored the experiences and attitudes of staff directly involved in the implementation of a new AI-assisted service delivery model. A recent survey of staff using AI-based clinical decision support tools found that their integration needs to be carefully considered to avoid poorly tailored and inappropriate tools^19^. This message was echoed by both medical professional and allied health screening staff, who highlighted the importance of involving end-users in the development and implementation of new models of care. It was clear that those involved saw value in providing eye screening in non-ophthalmic settings to improve DR detection rates and access to screening, reducing the burden placed on ophthalmology services, and enhancing job satisfaction by enabling staff to learn new clinical skills. Although the process of training and image grading was described as quick and easy, some staff found the setup of the system (camera, laptop, and printer) and the collection of data to be onerous. This was not surprising given that data collection routinely took place in ad-hoc spaces (shared waiting areas or office spaces), and research consent processes were lengthy. Concerns such as lack of confidence in explaining results to patients can be addressed by providing screening staff with further in-depth training relating to DR and its complications, as well as other common blinding eye diseases that may be detectable during retinal screening. Further, there remains a paucity of regulatory and legal guidance in Australia for AI-based systems^41^. In particular, the apportion of legal liability for possible incorrect diagnoses generated by these systems is unclear, with subsequent lack of medicolegal clarity potentially hindering uptake in real-world clinical practice^32^. Most recent regulatory guidance for software as a medical device (SaMD) issued by the Australian Therapeutic Goods Administration explicitly excludes AI-based systems, with AI-specific advice planned in separate future guidance^42^.

The current study has several major strengths. Firstly, the unique mixed methods study design revealed new insights into the real-world implementation of an AI-assisted DR screening model in diverse healthcare settings, from the perspective of both patients and clinicians. Secondly, our AI system utilised an offline solution which is not dependent on an internet connection. This feature is important for use in rural and remote regions where internet connectivity may be limited. Thirdly, more than one retinal camera model was utilised across study sites. This shows that the AI algorithm is transferable to different camera models with varying outputs and pixel quality. Lastly, the prospective mixed methods study design provided us with greater scope to investigate and contextualise both patient and staff experiences with this screening model.

Several limitations must also be considered. Firstly, participation rates were low, leading to a relatively small sample size compared to other studies^12^. This is possibly because participants were recruited from medical clinics and are already likely to be engaged in their healthcare, with the most common reason for declining to participate in this study being a recent eye test. The smaller sample size makes it difficult to assess the true impact of such a service in terms of disease detection rates and adherence to referral. Secondly, it is possible that patient and clinician participants who agreed to take part in this study had a greater existing interest in this technology, leading to potential positive selection bias in the feedback received. Despite this possible bias, feedback received was both positive and negative, adding significant qualitative colour to our findings (detailed in our Supplementary Tables). Thirdly, gold standard seven-field stereoscopic retinal photography and OCT were not utilised to determine the reference standard. The use of these gold standard imaging protocols is likely to have led to variations in the detection of referable DR by manual graders. Fourthly, clinic location was used as a proxy for indigenous status making it possible that participants attending endocrinology clinics may have identified as indigenous. Lastly, a thorough cost-effectiveness analysis of this model was not performed. Demonstrating the economic benefit of AI screening will be important for guiding policy change and stakeholder confidence. Differences in retinal camera costs, screening staff, clinic locations, and the inclusion of participants with pre-existing DR precluded our ability to make accurate cost estimates at an individual or population level compared to current screening practices.

The real-world implementation of an AI-assisted DR screening model is accurate and accepted by patients attending endocrinology and Aboriginal medical service clinics. Clinicians and screening staff involved responded positively to implementing such a service and found it to be quick and easy to use. To optimise compliance, and end-patient and public health benefit, future deployments of AI-assisted screening models in routine clinical practice require consideration of the downstream referral pathways for patients identified with DR.

## Supplementary Information

Supplementary Information.
