# Supplementary material for: Real-world﻿ artificial intelligence-based opportunistic screening for diabetic retinopathy in endocrinology and indigenous healthcare settings in Australia
Source: Sci Rep. 2021 Aug 4;11:15808. doi: 10.1038/s41598-021-94178-5 (PMC8339059; doi:10.1038/s41598-021-94178-5)
Supplement: Supplementary file 1 — Supplementary Information. [file 41598_2021_94178_MOESM1_ESM.pdf]

Supplementary Tables

Supplementary Table 1. Aspects of the AI screening model that worked well

	**Medical Professionals (n=3)**		**Allied Health Staff (n=5)**	
**Theme**	**Freq**	**Example quote**	**Freq**	**Example quote**
Easy to use	2	"Operationally it ran very smoothly"	12	"I actually found it quite easy"
Upskilling staff	10	"An advantage for us here at the hospital in terms of being involved in this project has been the upskilling of some of our nursing staff that have been taught how to use the retinal camera"	4	"I enjoyed using it actually. It's a good little tool"
Prompt feedback of results to patients	1	"To inform the patient better on what's happening next and also to include them and build their capacity"	8	"They (patients) were so happy that they actually got something tangible to take away with, or show their doctor as well"
Report easy to interpret	0	-	9	"It's like a red, green, orange system. It's got green means good and red means bad. To explain that to a patient is actually quite easy to do using the colour system"
Technical support	4	"People like […] come across, they know who to talk to"	4	"On occasions that I had [….] with me that was helpful when people's eyesight wasn't up to standard and that, sort of thing"
Quick system	0	-	6	"The screening itself only takes two seconds which was wonderful"
Improves efficiency of ocular care	2	"It (AI system) makes it all more of a one stop shop for getting things done"	3	"I thought it was fabulous and having it there as a bit of a one stop shop for patients"
Reduces patient burden	1	"Doing it on the spot, relatively quickly without mydriatics"	3	"Oh, can I drive afterwards – and I said yes no worries, I'm not putting any eye drops in, so that worked well"
Report ease of interpretation (patient)		-	3	"The patients were quite happy when they got to see whether it was red, green or amber, as in their diabetic retinal standard"
Identify multiple eye conditions	1	"I just applaud the efforts to be broad in the entry of multiple eye conditions. Not just diabetes. It was an impressive part of the tool"	1	"It's not only checking for diabetes it's checking for macular degenerates. It's checking for a multitude of

				different eye diseases which was really great”
Reliable	1	“We only had one or two that showed up positive results…one was a new finding which we subsequently got confirmed”	0	

* Frequency = number of times theme reported by participants

Supplementary Table 2. Challenges experienced with AI screening model

	**Clinician (n=3)**		**Staff (n=5)**	
**Theme**	**Freq**	**Example quote**	**Freq**	**Example quote**
Research procedures	7	“Because it's a prototype and it's a pilot study there's a lot of barriers to its use. And there is a time-consuming consent process required”	18	“But just I think the amount of paperwork that came with the actually screening. Filling out all the paperwork and all the questions”
Use of camera (patient-related factors)	0		13	“I found it really difficult at times and some people’s eyes, the pupil just wasn’t big enough. There was one lady who I had to say, I can’t take your photo because your pupil wasn’t dilated enough”
Knowledge (interpreting photos/reports)	0		9	“I didn’t completely understand the photos and the report and what they were meant”
Transfer of images	3	“So, photographs have to be done, loaded onto a USB in a format that we don't normally do. And then be transferred to another computer to use the software.”	4	“We sort of did have to - you know you take the photo on the OCT or the retinal camera and then you had to save it on a USB and then take it over to the laptop and pop it in”
Difficulties with camera (equipment)	1	“I think it was alignment was tricky sometimes. There were occasions when it just failed, I think and had to be done again or failed and had to be abandoned.”	6	“Sometimes I did find it hard lining it up and then I’d be – sorry I’m really sorry, it’s not a great photo. I don’t know whether an Ophthalmologist can evaluate those photos or not”
Opportunity to practice	0		7	“I forgot how to use it after a while because I didn't use it enough”
Staffing resources	1	“Getting the staff trained (is a challenge)”	5	“Because the clinic was just so busy doing the AI software on

				top of already the other tests that we were doing was”
Software upgrade	0		6	“When I downloaded the software for the upgraded version it went a bit funny and I just had no idea how to sort it all out”
Room availability	2	“Finding space where the thing could be safely stored”	2	“The set up across the road wasn’t all that great because the room was a bit small. There was a lot more of everything in the room”
Technical (report)	0		3	“The computer was spitting out the exact same report to everyone”
Financial resources	2	“Paying for staff.”	0	

* Frequency = number of times theme reported by participants

Supplementary Table 3. Key factors to integrating AI systems into clinic settings

	**Medical Professionals (n=3)**		**Allied Health Staff (n=5)**	
**Theme**	**Freq**	**Example quote**	**Freq**	
Efficient and user-friendly setup of AI system	3	“It needs to be automated and inbuilt in the camera hardware. If at all possible, through hardware or software integration, to avoid an extra step”	17	“So, I was thinking about maybe having the AI algorithm somehow uploaded onto the retinal camera. Maybe just not having that second computer”
Linked with a referral pathway	10	“We find something that needs to be acted on maybe to trigger a designated referral pathway that we might have buddied up with one of the ophthalmologist close by here to say “*This is something we picked up in the clinic, can you see them the next week, they might need laser therapy or an injection?”*.	6	“They would need to just know where their photo is going and how long that that's going to take to get the result and what's going to happen from there”
Staff training	0		11	“If I continued with it, I’d like some more specialised training to be able to troubleshoot and perhaps give more detailed, you know, answers to questions

				because people want to know”
Evidence for reliability/validity	8	“I think first of all establishing the reliability of the technology which is what we are about at the moment in the real world.”	3	“Evidence-based practice can encourage more people to actually have confidence in it”
Resources (adequate staffing)	0		8	“You know, having a rotated roster but one person needs to be responsible and accountable for that (AI system) and that alone, not running the rest of the clinic”
Clinician support	0		7	“We need to definitely let the Doctors know, let the Endocrinologists know so that they are on board and they can see how it works”
Financial resources	6	“Working out how to fund it which is coming down to the cost of the staff, the cost of the machinery and its maintenance”	0	
Free service	2	“We rely on patients paying their way private or going to the Eye and Ear Hospital, both of which are less than satisfactory for most of them”	4	“It needs to be a free service. Especially if we’re targeting those population that can’t afford to have this service. And I think they’re the ones who can benefit most out of it.”
Staff with eye health training	2	“We still need someone with some training in eye health such as an optometrist would be an excellent person to work in conjunction with this system”	4	“Someone with knowledge -they will not pick up that there's an issue going on. And then that person may walk away with - or come back and have clear or good and they've been given the green light when in actual fact it might have been a red light or an amber light”
Providing integrated eye health care	4	“It’s trying to get those last few by making the screening integrated into the rest of their care. So, they don’t have to make a separate appointment to	2	“But I think if you wanted this AI or the testing to be as part of clinic, I think scheduled appointments would be a better way to

		see a separate doctor, pay $150, that's the barrier"		go…as part of routine care"
Simplify report	0		4	"The grading, the M1, M0. Sometimes if we can speak to positive, negative, ungradable, like printed in words, I think it would be better for the patient. Because they always, always ask me "what does that mean"?""
Advocacy and marketing of AI system	1	"Making the case that it's a service that Eastern Health need to provide, and we've been neglecting our duty to our patients up to this point"	3	"Letting them (patient) know in advance that it's there and they can have it done"
Technical support	1	"You know that there's somebody you could ring up if there's a problem, and that they're not coming in."	2	"Just technical support along the way"
Real-time feedback to patients	3	"It's very important that a patient is informed on the spot that there's something requiring referral and urgent to be seen"	0	

* Frequency = number of times theme reported by participants

Supplementary Table 4. Value of AI screening for eye disease

	**Medical Professionals (n=3)**		**Allied Health Staff (n=5)**	
**Theme**	**Freq**	**Example quote**	**Freq**	**Example quote**
Enhance screening, detection, diagnosis	6	"If it becomes embedded in a public hospital diabetics clinic…the routine screening of eyes would improve"	10	"If you're preventing eye diseases, that's going to be overall a benefit financially for healthcare in general"
Reduce burden on eye HP's and improve effective utilisation of eye HP's	7	"We're all so busy, that it'd be good maybe to use our abilities in other areas rather than the routine practice of looking at things"	1	"I think that it would highlight that people who don't need as much follow-up - so say someone's got a green outcome, or a green report, we don't need to see them for 12 months. So, then that eliminates that part of their healthcare for 12 months unless something pops up."
Triage tool	4	"It would help arrange the treatment that's	2	"It would highlight that people who don't need as

		required and reduce non-attendance at future treatment.”		much follow-up …some get the red report a lot more care needs to be put into that person so they would need see more people”
Improve patient care	3	“It would just improve patient understanding and therefore overall improve patient healthcare.”	3	“It gives someone an opportunity who may not be able to get to the Ophthalmologist or Optometrist.”
Upskill health professionals	2	“Some of our nursing staff that have been taught how to use the retinal camera ...and can see that they’re also contributing to the bigger picture of things in terms of diabetic care... “	0	
Useful tool in regional areas	1	“Extremely useful is maybe in remote communities outside of a metropolitan area where a photograph can be taken and a report’s generated.”	0	
Cost savings	1	“It would cost you a lot more having an Ophthalmologist on your staff.”	0	

* Frequency = number of times theme reported by participants
